# Continuous Depletion of Tetrahydrocannabinol From *Cannabis* Extract Through Simulated Moving Bed Chromatography Using Green Mobile Phase

**DOI:** 10.1002/jssc.70175

**Published:** 2025-05-22

**Authors:** Greta Compagnin, Chiara De Luca, Chiara Nosengo, Giorgia Greco, Martina Catani, Alberto Cavazzini, Yannick Krauke, Simona Felletti

**Affiliations:** ^1^ Department of Environmental and Prevention Sciences University of Ferrara Ferrara Italy; ^2^ Department of Chemical, Pharmaceutical and Agricultural Sciences University of Ferrara Ferrara Italy; ^3^ KNAUER Wissenschaftliche Geräte GmbH Berlin Germany; ^4^ Council for Agricultural Research and Economics Rome Italy

**Keywords:** cannabinoids, *Cannabis sativa* L., green mobile phase, simulated moving bed, THC depletion

## Abstract

The interest toward greener and more sustainable chemical processes is constantly growing, especially in chemical industries, where there is an urgent need to reduce the waste on one hand, and to replace not environmentally friendly organic solvents, on the other.

One of the current challenges in industrial processes is the depletion of the psychoactive cannabinoid tetrahydrocannabinol (THC) from hemp extracts. Indeed, current legislations impose that THC in hemp products must meet defined concentration limits or, in some cases, it must be completely removed. In the present study, the continuous depletion of THC has been performed through simulated moving bed chromatography operated with a fully green mobile phase, made of water and ethanol. This process permits to overcome the limits and drawbacks of available purification methods, based on toxic solvents and traditional single column chromatography.

## 1 Introduction

Preparative liquid chromatography is the most common technique used in industry to purify target compounds from more or less complex matrices [1, 2]. Conventional set‐up consists of a single column, which is commonly addressed to as batch chromatography. It is based on a discontinuous process where a defined volume of analyte is injected, then separated and collected. Despite its ease of use, simple setup, and user‐friendly interface, it shows some disadvantages, including long processing time and environmental issues given the large amount of solvent waste produced per gram of purified product. This poses a concern about the sustainability of batch purification processes, which urgently needs to be addressed. Indeed, in recent years, much interest and efforts have been devoted to obtain and develop innovative processes able to provide similar or higher performance with respect to common procedures but with reduced environmental impact.

Concerning chromatographic methods, the concept of the three *Rs* can be applied to favour the green transition, that is, Reduce, Replace, and Recycle. Reduce indicates the need of decreasing the waste produced; Replace implies that toxic solvents must be changed with greener alternatives; and Recycle means that solvents should be reused.

Despite nowadays the use of green solvents in chemical industry and processes is constantly gaining more and more importance [3], as demonstrated by the increasing number of publications about the topic, the shift to methods that enable waste reduction and solvent recycling is more challenging. This is essentially due to the fact that multicolumn platforms should be used for the purpose. These are characterized by a more complex setup if compared to single column instruments, which consequently implies the need for highly qualified personnel for their operation and management. Nonetheless, multicolumn chromatography is attracting interest from companies that are increasingly introducing these techniques in their daily work, especially for large‐scale TIDES (oligonucleotides and peptides) purification [4, 5, 6, 7, 8, 9].

Multicolumn preparative chromatography represents the optimal choice to simultaneously achieve the goals imposed by the three *Rs*. Indeed, conversely to single column batch, it is a continuous process where the feed is continuously injected into the system and continuously purified and isolated. Moreover, the possibility of reinjecting some fractions, as it happens for multicolumn countercurrent solvent gradient purification (MCSGP), or of internally recycling the mobile phase, as for closed‐loop simulated moving bed (SMB), permits to obtain a considerable reduction of solvent waste and purification time. These processes are particularly advantageous for industrial scopes where the highthroughput continuous production of purified target is needed. In this context, one specific case is given by the removal of the psychoactive cannabinoid (−)‐Δ^9^‐tetrahydrocannabinol (THC) from *Cannabis sativa* L. extracts. Indeed, due to current legislations, which impose strict THC concentration limits, or in some cases the complete absence of THC, processes able to reduce or completely deplete the THC content in the final product are highly needed [10, 11]. Dilution, crystallization, and fractional distillation can be used for the purpose. However, their application to industrial and commercial scale would be complicated due to costs, loss of products, and difficulties related to the similar structures and boiling points of cannabinoids [12]. Preparative liquid chromatography, on the other hand, represents the most promising technique thanks to its enhanced selectivity and efficiency, compared to other methods [13, 14, 15, 16, 17]. In literature, THC‐depletion is mainly described by liquid chromatography either under reversed phase (RP) or normal phase (NP) conditions using acetonitrile/water mixtures or *n*‐hexane/ethanol as mobile phases and traditional C18 or silica columns as stationary phases [12, 18, 19, 20, 21]. Despite being a very well established technique for the separation and quantification of cannabinoids in analytical scale [18, 22, 23, 24, 25, 26, 27], RPLC operated with aqueous mobile phases has some limitations when applied to preparative scale. Indeed, cannabinoids, being lipophilic molecules, have very poor solubility in water (less than 1 µg/mL) [15], forcing to perform their extraction and storage with organic solvents. This does not represent an issue when analytical chromatography is concerned, due to the low analyte injection volume (few µL). On the other hand, in preparative chromatography larger injection volumes (from mL to L) are often used to maximize bed utilization and throughput. In this scale, the content of organic modifier contained in the sample must be low enough to allow the target product adsorption on the stationary phase. Usually, an amount of < 20% of organic modifier should fit for this scope, even though it strictly depends on the feed of interest, and it is difficult to generalize. This is in contrast with the need of a high content of organic solvent necessary in the feed to dissolve cannabinoids, which on the other side, forces to decrease the injection volume to a few microlitres to favor analyte retention. This prevents the known advantages of preparative chromatography and requires that more repetitions of the same purification are subsequently performed to achieve the desired amount of pure product, with a consequent increase in solvent consumption.

To overcome the RP preparative chromatography issues, some authors of this paper, described a THC removal process under NP elution mode using SMB [28]. In this case, in addition to solving solubility problems (e.g. CBD solubility in hexane is > 100 mg/mL), a continuous system like SMB allows to reduce solvent consumption and increase productivity. However, all these techniques, operated in both RP and NP elution modes, do not stand out in greenness. Indeed, acetonitrile (ACN) is ranked as a “problematic” solvent on par with heptane in terms of greenness and toxicity, since it has important environmental and health implications [9, 29, 30, 31]. Hence, the use of alternative, greener solvents, and techniques is of utmost importance. In this field, the present work advances the field of green chemistry describing for the first time a fully green and sustainable method based on multicolumn chromatography for the continuous THC‐depletion of *Cannabis* extracts. The purification process is based on SMB technology operated under RP elution mode, with a fully green mobile phase made of 20%/80% water/ethanol. Ethanol (EtOH) is indeed classified as a fully “recommended” solvent, with no negative effects on both environment and humans [31, 32]. To access the advantages of multicolumn techniques over traditional single column processes, the common procedure, described in various publications reported in literature [15, 33, 34, 35, 36, 37, 38], is to compare the SMB performance to batch operated under the same experimental conditions.

## 2 Simulated Moving Bed Theory

SMB is a multicolumn continuous countercurrent chromatographic process applied for binary or pseudo binary separations in different areas, from the petrochemical to the pharmaceutical field [15, 33, 34, 35, 36, 37, 38]. In SMB the feed mixture is separated into two fractions (the less retained raffinate and the most retained extract) through the opposite direction of mobile and stationary phase flows. This is achieved through the use of switching valves, operated synchronously at a defined switching time, which simulates the movement of the stationary phase. In SMB the feed is continuously injected into the system made of a defined number of identical chromatographic columns, from 4 to 16, depending on the case [39, 40].

**FIGURE 1:**
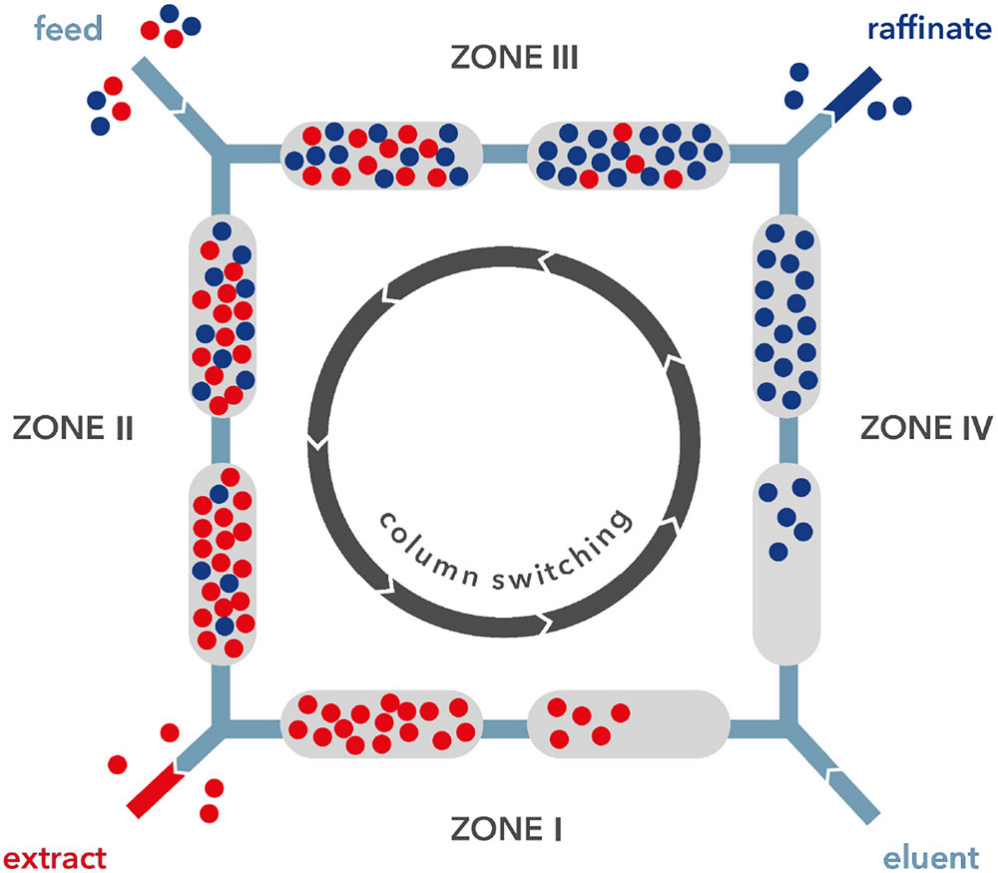
Schematic configuration of the 8‐column‐SMB system for binary separations [37]. Blue points: raffinate fraction. Red point: extract fraction.

The SMB system is subdivided into four zones (*j* = I, II, III, IV), as shown in Figure 1: Zones II and III are directly responsible for the chromatographic separation, with the feed inlet in between; while, Zone I and IV are defined buffer zones, because the mobile phase inlet is located between them and permits the complete regeneration of the solid (Zone I) and liquid phase (Zone IV). The system is characterized by four internal flow rates, $Q_{j}$, that is, the flow rate in each zone, and four external flow rates, which represent the flow rates supplied by pumps ($Q_{D}$= desorbent, $Q_{E}$= extract, $Q_{F}$= feed, and $Q_{R}$= raffinate). These eight flow rates are directly related to each other through the following fluid phase balances [40]:

(1a) $$Q_{I}=Q_{IV}+Q_{D}$$

(1b) $$Q_{II}=Q_{I}-Q_{E}$$

(1c) $$Q_{III}=Q_{II}+Q_{F}$$

(1d) $$Q_{IV}=Q_{III}-Q_{R}$$

where, for proper setup of the SMB system, the optimal flow rates in each zones must be carefully selected [33, 39, 40, 42, 43]. One of the most widely established method for the identification of the most suitable flow rates reported in Equation (1) is the so‐called Triangle theory [39, 44, 45, 46]. This method defines a complete separation region by plotting $m_{j}$ relative to Zones II and III. $m_{j}$ is defined as the ratio of liquid ($Q_{j}$) to solid ($Q_{s}$) flow rates in zone *j* [44]:

(2) $$m_{j}=\frac{Q_{j}}{Q_{s}}=\frac{Q_{j}\;t^{*}\;-\;V_{col}\varepsilon_{t}}{V_{col\;}\left(1\;-\;\varepsilon_{t}\right)}$$

where $t^{*}$ is the switch time of the SMB unit, $V_{col}$ the geometrical column volume and $\varepsilon_{t}$ the column total porosity.

**FIGURE 2:**
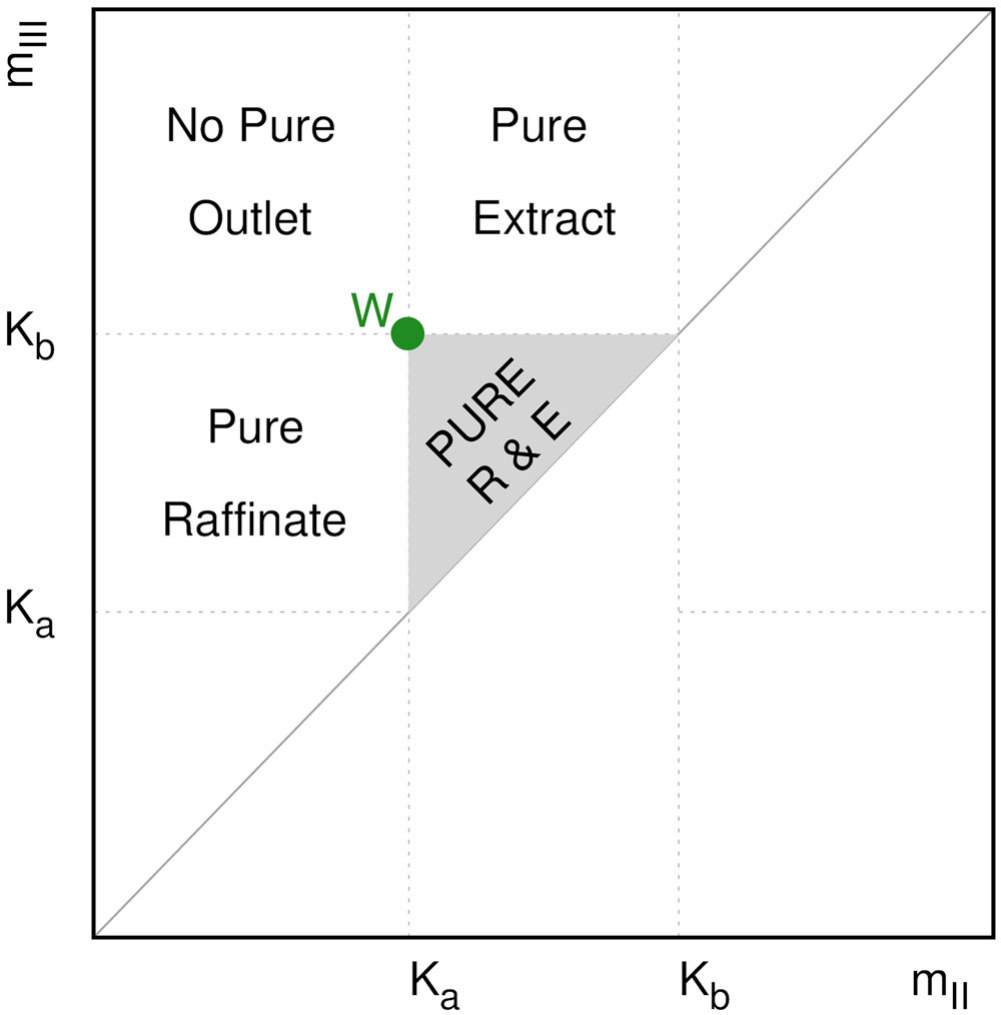
Representation of Triangle theory for linear isotherms considering a binary separation of *a* and *b* components, with *b* the more retained one. Pure E and R: separation region, where both pure raffinate and extract are collected. Pure raffinate: region where pure raffinate is obtained. Pure extract: region where pure extract is obtained. No pure outlet: region where no pure fractions are obtained, that is no separation region.

For linear adsorption isotherms, the binary mixture can be completely separated only if the following inequalities are satisfied:

$$K_{b}\leq m_{I}$$

$$K_{a}<m_{II}\leq K_{b}$$

(3) $$K_{a}\leq m_{III}\leq K_{b}$$

$$m_{IV}\leq K_{a}$$

where $K_{a}$ and $K_{b}$ are the Henry constants of raffinate and extract, respectively. In the triangle (see Figure 2), the point with the highest achievable separation performance, in terms of purity and productivity, is the summit *W* in which $m_{II}=K_{a}$ and $m_{III}=K_{b}$. Outside the triangle it is not possible to obtain the two pure outlets (raffinate and extract) at the same time. Indeed, in this region, only one pure outlet (either raffinate or extract) or both impure outlets can be achieved. For a more detailed description of the triangle theory, the reader is referred to [39, 44, 45, 46].

## 3 Performance Parameters and Green Metrics

To evaluate the performance of a purification method different metrics can be taken into account: purity, recovery, and productivity [39, 47].

The purity of the target, eluted either from raffinate or extract fractions at the cyclic steady state, is defined as the ratio of the area of the target component ($A_{i}$) to the total area of the chromatogram ($A_{total}$):

(4) $$P_{i}\left(\%\right)=\frac{A_{i}}{A_{total}}\cdot 100$$

The recovery is defined as the ratio of the mass of the target component collected ($m_{i}$) at the two outlets to the total amount of this species present in the feed ($m_{i,total}$):

(5) $$Rec_{i}\left(\%\right)=\frac{m_{i}}{m_{i,total}}\cdot 100$$

The productivity represents the ratio of the amount of target analyte ($m_{i}$) collected per unit of run time (time) and per total volume of stationary phase, defined as the geometrical column volume multiplied by the number of columns in the system ($V_{col\;}$ *·* $n_{col\;}$):

(6) $$Productivity\;\left(g/h/L\right)=\frac{m_{i}}{time\;\cdot \;n_{col\;}\cdot \;V_{col\;}}$$

where these parameters, as already stated, define the quality of the chromatographic step from the performance viewpoint, neglecting the environmental impact. Although various approaches have been proposed to measure the sustainability of processes and products, there is still no unified set of metrics [49] that can be applied independently from the chemical process. As an example, one of the simplest metrics for assessing the sustainability of a process is the environmental factor (E‐factor), which measures the amount of waste produced based on the amount of purified product [50] (E‐factor = $m_{waste}/m_{product}$). However, in cases where no waste is generated, thanks to the internal recycling of solvents, such as SMB operated in closed‐loop operation mode, the E‐factor is not an effective metric for assessing the environmental impact of the process, hence other parameters need to be considered.

A useful metric is the (total) solvent consumption, defined as the volume of solvent used ($V_{tot\;}$) per mass of recovered target component ($m_{i\;}$):

(7) $$Solvent\;Consumption\;\left(L/g\right)=\frac{V_{tot\;}}{m_{i\;}}\;\;$$

Another metric for assessing the greenness is the AGREE (Analytical GREEnness) calculator [41]. Based on the 12 principles of green chemistry, it generates a greenness score ranging from 1 (highest level of greenness) to 0 (lowest level of greenness). In this study, we only considered eight parameters, tailor made to access the greenness of preparative chromatographic processes (see Supporting Information for more details): (1) Position of analytical device; (2) Automatic and Continuous or Discontinuous process; (3) Solvent Consumption; (4) Productivity; (5) Purity; (6) Recovery; (7) Waste; (8) Solvent Toxicity.

## 4 Experimental Section

### 4.1 Chemicals and Solvents

Cannabinoids standard solutions (1 mg/mL in methanol), namely cannabidiol (CBD), THC, and cannabichromene (CBC) were purchased from Cerilliant (Round Rock, Texas, USA). Orthophosphoric acid (85%), HPLC grade solvents, including ACN and EtOH were from Sigma–Aldrich (St. Luis, MI, USA). The sample was an ethanolic extract from a decarboxylated *Cannabis sativa* variety rich in CBD. Sample was filtered with 0.2 µm PTFE filter prior to injection.

### 4.2 Analytical Analysis

The characterization of the CBD‐rich extract and the off‐line measurements were performed on AZURA HPLC system

(KNAUER, Berlin, Germany) equipped with a binary pump, a column thermostat, an autosampler, and a photodiode array detector. Data acquisition, data handling, and instrument control were performed by ClarityChrom CDS software. Initial and final purity and concentration of the sample were accessed using a 150 × 4.6 mm Eurospher II C18P column packed with 3 µm fully porous particles (KNAUER, Berlin, Germany). Mobile phases were a phosphate buffer solution at pH = 2.2 and pure ACN. The gradient program was set as follows: 0–7 min 75% ACN, 7–17 min from 75% to 90% ACN, 17–19 min 90% ACN, 19–22 min 75% ACN [42]. The wavelength was set at 228 nm. Injection volume was 10 µL. Calibration was performed using cannabinoid standards with known concentrations, ranging from 0.5 to 100 µg/mL.

### 4.3 Simulated Moving Bed

The SMB purification has been performed on a stainless steel AZURA Lab SMB System (KNAUER, Berlin, Germany) equipped with seven 8‐multiposition valves, four pumps, and two mass flow controllers (Mini CORI‐Flow). The system was controlled by PurityChrom, MCC software. Preparative method was scaled up from a Eurospher II 150 × 4.6 mm C18 column packed with 15 µm fully porous particles (KNAUER, Berlin, Germany) to a 250 × 8.0 mm C18 column packed with 15 µm fully porous particles from the same manufacturer (data not shown), using a green mobile phase made of 80%/20% EtOH/H_2_O and a flow rate of 4 mL/min in Zone I. A set of eight 250 × 8.0 mm (15 µm) C18 columns were used with a 2:2:2:2 configuration. Different feed velocities ($Q_{feed}$) have been tested, from 0.04 to 0.08 mL/min.

### 4.4 Batch Chromatography

Batch purification was performed on KNAUER preparative HPLC system equipped with a binary pump (50 mL head pump), one semi preparative 3 mm UV flow cell and a fraction collector Foxy R1, using the same column (250 × 8 mm), mobile phase (EtOH/H_2_O 80%/20%), and flow rate (4 mL/min) as the SMB separation in Zone I. Run time was 12 min. Data acquisition, data handling, and instrument control were performed by PurityChrom CDS software. The feed injection volume was limited to 100 µL for the reasons explained in the Introduction section.

For a fair comparison with the SMB purification, the batch chromatogram was cut only into two fractions, corresponding to SMB raffinate and extract.

## 5 Results and Discussion

**FIGURE 3:**
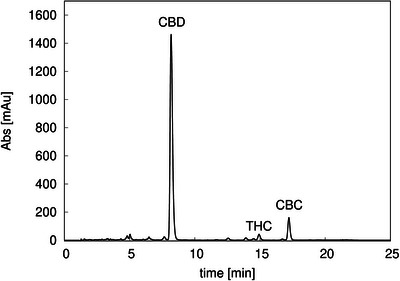
Analytical chromatogram of *Cannabis* extract obtained with gradient analytical method (see Section 4.2). CBC: cannabichromene, CBD: cannabidiol, THC: tetrahydrocannabinol.

First, the *Cannabis* sample was characterized using the analytical gradient method described in Section 4.2 in order to identify and quantify the cannabinoids of interest. The obtained chromatogram is shown in Figure 3, where the three main cannabinoids are CBD (11.7 mg/mL), THC (0.31 mg/mL), and CBC (0.50 mg/mL). CBD initial purity was 88%.

**FIGURE 4:**
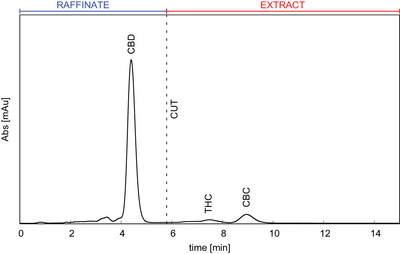
SMB starting chromatogram of *Cannabis* extract obtained with isocratic method 80%:20% EtOH/H_2_O on 250 × 8.0 mm C18 column packed with 15 µm fully porous particles Injection volume = 20 µL.

Once the optimal experimental parameters have been identified under analytical conditions (150 × 4.6 mm C18 column) in terms of mobile phase composition and flow rate, and the linearity of adsorption isotherms of the target has been verified by injecting increasing volume of feed up to 100 µL, the scale‐up to preparative conditions (250 × 8.0 mm C18 column) has been performed. The feed chromatograms obtained on the 250 × 8.0 mm column at a flow rate of 4 mL/min by injecting 20 µL for the SMB and 100 µL for the batch purifications are reported in Figure 4 and Figure S1, respectively. In both cases, the desired separation region, between CBD and THC peaks, is defined by the “CUT” line. The batch process has been designed in order to obtain only two fractions to mimic the outcomes of the SMB binary separation, so that batch Fraction 1 corresponds to SMB raffinate and batch Fraction 2 corresponds to SMB extract. Batch run time was set to 12 min to permit the elution of all the impurities.

From Figure 4 the SMB starting parameters are obtained and are listed in Table 1. CBD and THC retention factors were used to set the Henry constants of raffinate ($K_{a}$) and extract ($K_{b}$), respectively, knowing that $K_{i}=k_{i}\;/F$, with F the phase ratio.

**TABLE 1:** SMB starting parameters.

Parameter	Value
SMB configuration	2‐2‐2‐2
Number of columns	8
Column ID [mm]	8.0
Column volume [mL]	12.56
Particle size [µm]	15
Total porosity	0.57
$Q_{I}$ [mL/min]	4.0
$Q_{feed}$ [mL/min]	0.08
*t^∗^* [min]	8.34
$k_{CBD}$	1.42
$k_{THC}$	2.82
$K_{a}$	1.92
$K_{b}$	2.82

Raffinate and extract fractions have been analyzed off‐line after each cycle. This permits, on the one hand, to directly check the separation by verifying CBD recovery and concentration in the two outlets and, on the other hand, to determine when the steady state has been reached (i.e. when the composition of the eluate is constant). Moreover, SMB permits to reach a final product recovery up to 100% thanks to the continuous collection of the two fractions from the first cycle.

The analytical chromatograms of raffinate and extract are reported and compared in Figure S2. The results of the analysis of the raffinate from SMB and Fraction 1 from batch, that is, CBD‐rich outlets, listed in Table 2, showed that THC content was below the detection limit. This confirms that both techniques successfully depleted THC from the *Cannabis* sample, achieving similar CBD recovery rates (> 98%) and a *∼* 11% increase in CBD purity with respect to the initial crude sample, reaching a final purity of roughly 98%.

**TABLE 2:** Comparison between performance parameters of SMB and batch chromatography.

Method	Purity	Recovery	Productivity	Productivity	Solvent consumption
	(%)	(%)	*g_CBD_*/h/L	*mg_CBD_*/h	L/*g_CBD_*
SMB	97.80	98.76	0.57	56.85	3.29
Batch	97.99	99.03	0.47	5.85	41.05

The most significant differences between the SMB and batch processes consist in productivity and solvent consumption, both influenced by the type of chromatography (continuous vs. discontinuous) used. Since the *Cannabis* extract was an ethanolic solution, it was not possible to inject large volumes of feed in the batch process, otherwise compounds would have been eluted near the dead volume of the column with unacceptable resolution between CBD and THC due to the strong effect of EtOH [17]. As a consequence, this negatively impacts on both the final productivity and solvent consumption since subsequent injections and purification procedures need to be performed to purify a defined amount of CBD. As an example, 1 mg of CBD is obtained in roughly 10 min using 40 mL of solvent with batch chromatography, while only 1 min and 3 mL of solvent are necessary for SMB (at the steady state). This clearly indicates that, despite the more complex setup and cost of the SMB system, it permits to dramatically reduce solvent consumption of more than 90% and to increase productivity (Equation 6) of 21% compared to batch chromatography. Moreover, if productivity is expressed in absolute terms, that is, without considering the total column volume, SMB provides roughly 10 times larger values than batch (56.9 vs. 5.9 mg/h), as shown in Table 2. These advantages for the SMB system are given by the use of SMB in closed‐loop operation mode, where Zone IV is directly connected with Zone I, meaning that waste is not produced and no more solvent is injected because it is recycled after the first cycle, coupled to the possibility of continuously load the *Cannabis* ethanolic extract into the system. This is performed using a slow flow (< 0.1 mL/min) that doesn't affect analyte elution due to the balance existing between SMB flow rates (see Section 2). Indeed, the feed flow is “diluted” in Zone III with the mobile phase eluting from Zone II, whose flow rate is much larger (*∼* 2.2 mL/min), hence not influencing analyte retention.

In addition to the solvent consumption, the greenness of the two processes was determined using a modified AGREE metric. AGREE generates a number from 0 to 1, by evaluating a set of parameters according to the process considered, where the closer the number to 1 the more that process is green [41].

**FIGURE 5:**
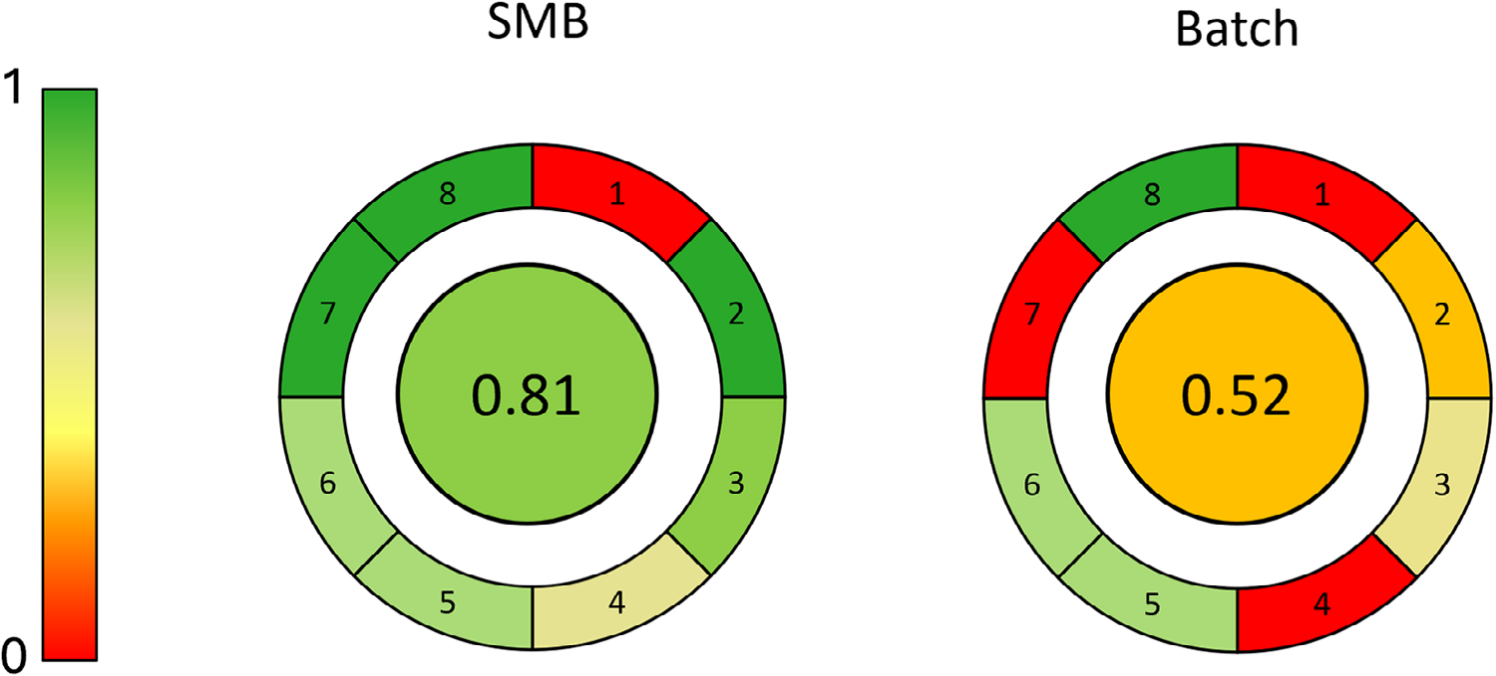
Representation of AGREE results. Numbers from 1 to 8 indicate the parameters used to generate the greenness value reported in the center of each circle diagram. The column chart on the left is the corresponding color scale for reference.

In this study, the parameters considered by AGREE were (see Supporting Information for a more detailed explanation): (1) Position of analytical device: in = 1, out = 0; (2) Automatic and continuous or discontinuous process: Automatic and continuous = 1, automatic and discontinuous = 0.5, manual and discontinuous = 0; (3) Solvent consumption: 0 mL/min = 1, 100 mL/min = 0; (4) Productivity: 100 mg/h = 1, 0 mg/h = 0; (5) Purity: 100% = 1, 0% = 0; (6) Recovery: 100% = 1, 0% = 0; (7) Waste: No = 1, yes = 0; (8) Solvent toxicity: green solvents = 1, toxic solvents = 0. The calculated parameters for SMB and batch purification processes are listed in Table 3. The results from AGREE calculations are shown in Figure 5, where SMB is confirmed as a greener technique for THC depletion from CBD rich *Cannabis* samples if compared to traditional batch chromatography. As it can be noticed, the most critical point in both cases is the first parameter, related to the position of the off‐line device for the analytical analysis of the fractions. Even if this step cannot be excluded from the purification procedure, it is worth mentioning that only an initial check of the fractions is required once the robustness and reproducibility of the purification method(s) are verified, leading to an additional saving of resources, solvents, and costs.

**TABLE 3:** AGREE parameters.

Parameter	SMB	Batch
1	0	0
2	1	0.5
3	0.97	0.59
4	0.57	0.06
5	0.98	0.98
6	0.99	0.99
7	1	0
8	1	1

## 6 Conclusions

Chromatography is moving toward more sustainable methods that can be directly applied for industrial scopes. In this regard, the present work is focused on a topic of great industrial importance as the depletion of the psychoactive cannabinoid THC from *Cannabis* extracts to make it easier their commercialization, following current legislations. In more detail, two techniques, namely single‐column batch and multi‐column SMB, have been evaluated and compared for the highthroughput THC depletion. Both processes have been operated under RP elution mode using a fully green mobile phase made of a mixture of EtOH and water, permitting a first reduction of the environmental impact of preparative chromatography if compared to currently available method. Moreover, it has been highlighted and demonstrated how the use of a continuous technology, as SMB, can offer significant advantages in terms of process automation, reduction of solvent waste and solvent consumption, overall leading to an increase in productivity and sustainability.

## Author Contributions

**Greta Compagnin**: formal analysis, investigation, writing–original draft. **Chiara De Luca**: data curation, formal analysis, supervision. **Chiara Nosengo**: data curation, formal analysis. **Giorgia Greco**: methodology, funding acquisition, resources. **Martina Catani**: methodology, visualization. **Alberto Cavazzini**: funding acquisition, supervision. **Yannick Krauke**: methodology, conceptualization, writing–review and editing. **Simona Felletti**: conceptualization, supervision, writing–review and editing.

## Conflicts of Interest

The authors declare no conflicts of interest.

## Supporting information



Supporting Information

Supporting Information

Supporting Information

## Data Availability

The data that supports the findings of this study are available in the Supporting Information of this article.
