# Supplementary figures and images for: Continuous Depletion of Tetrahydrocannabinol From Cannabis Extract Through Simulated Moving Bed Chromatography Using Green Mobile Phase
Source: J Sep Sci. 2025 May 22;48(5):e70175. doi: 10.1002/jssc.70175 (PMC12096815; doi:10.1002/jssc.70175)

Fraction 1

Fraction 2

Abs [mAu]

CBD

CUT

CBC

THC

0

2

4

6

8

10

12

14

time [min]

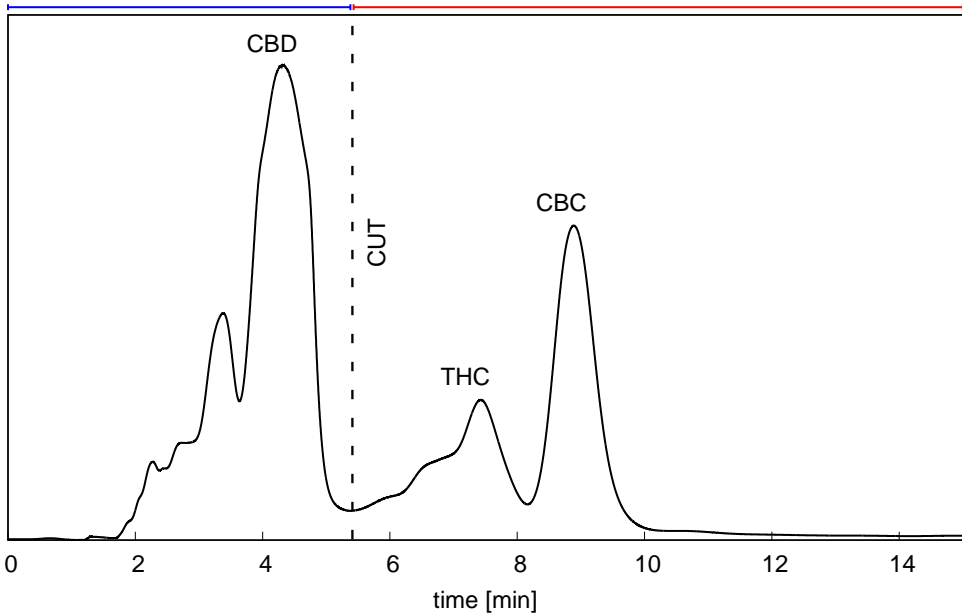

Supplement: Supplementary file 1 — Supporting Information [file JSSC-48-e70175-s002.pdf]

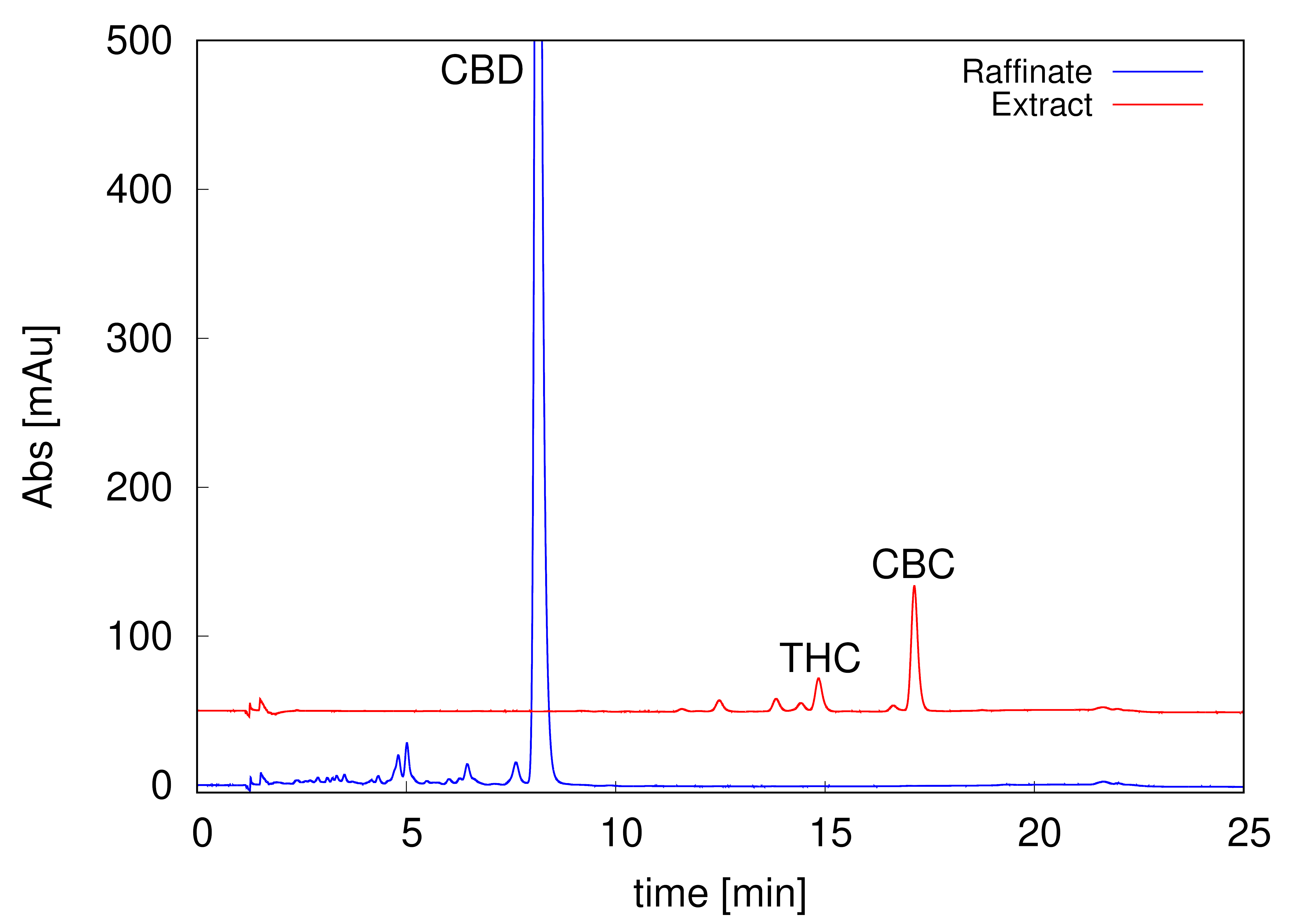

Supplement: Supplementary file 2 — Supporting Information [file JSSC-48-e70175-s001.jpg]
