# Supplementary material for: Continuous Depletion of Tetrahydrocannabinol From Cannabis Extract Through Simulated Moving Bed Chromatography Using Green Mobile Phase
Source: J Sep Sci. 2025 May 22;48(5):e70175. doi: 10.1002/jssc.70175 (PMC12096815; doi:10.1002/jssc.70175)
Supplement: Supplementary file 3 — Supporting Information [file JSSC-48-e70175-s003.docx]

**Supplementary Material**

**Continuous depletion of tetrahydrocannabinol from Cannabis extract through simulated moving bed chromatography using green mobile phase**

Greta Compagnin^1^, Chiara De Luca^2^, Chiara Nosengo^2^, Giorgia Greco^3^, Martina Catani^2^, Alberto Cavazzini^2,4^, Yannick Krauke^3^, Simona Felletti^1^

^1^Department of Environmental and Prevention Sciences, University of Ferrara, Italy, Ferrara

^2^Department of Chemical, Pharmaceutical and Agricultural Sciences, University of Ferrara, Italy, Ferrara

^3^KNAUER Wissenschaftliche Geräte GmbH, Germany, Berlin

^4^Council for Agricultural Research and Economics, Italy, Rome

1. **CHROMATOGRAMS**

**
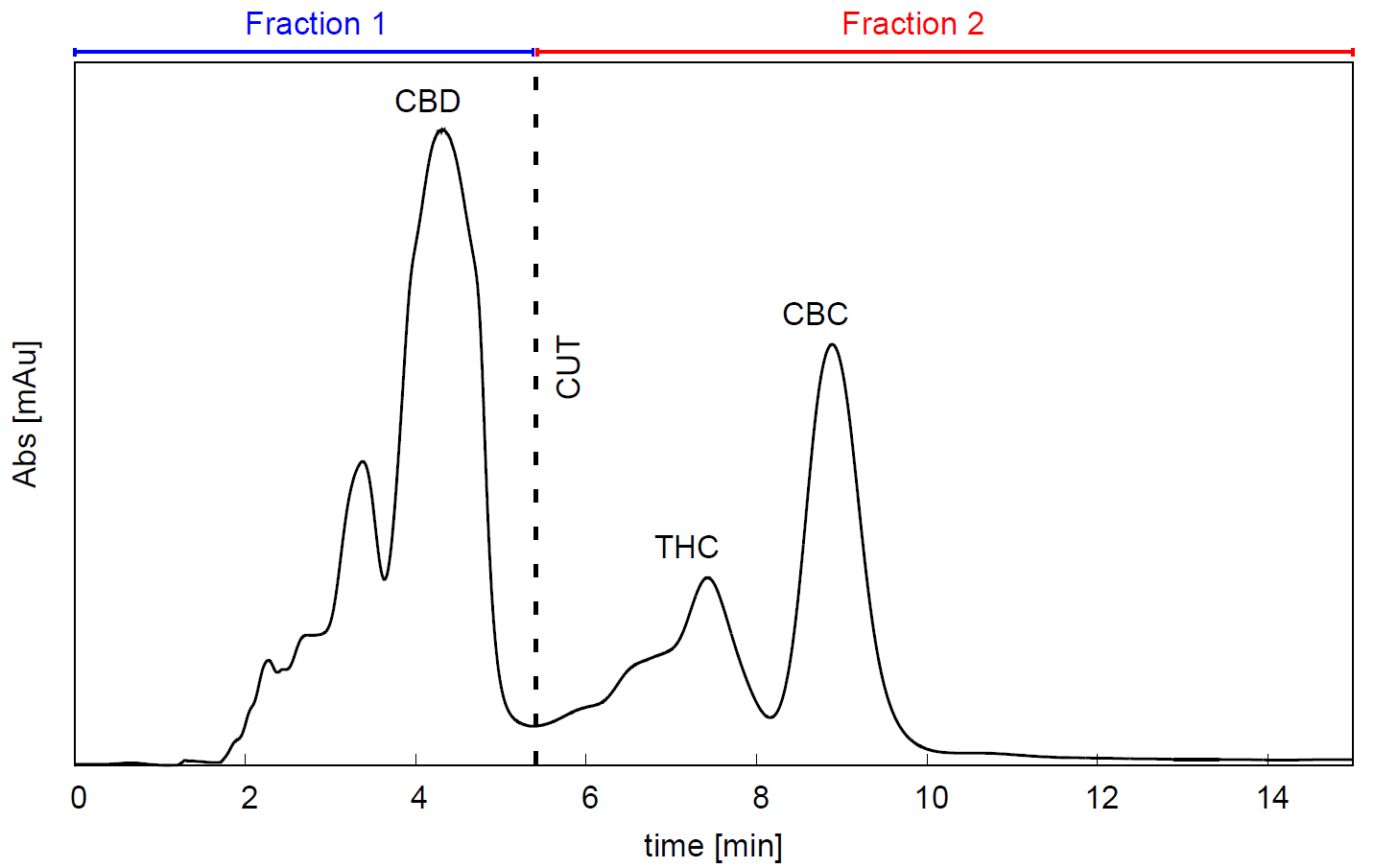
**

FIGURE 1: Batch chromatogram of *Cannabis* extract obtained with isocratic method 80/20 % EtOH/H_2_O on 250 *×* 8.0 mm C18 column packed with 15 *μ*m fully porous particles. Injection volume = 100 *μ*L.

**
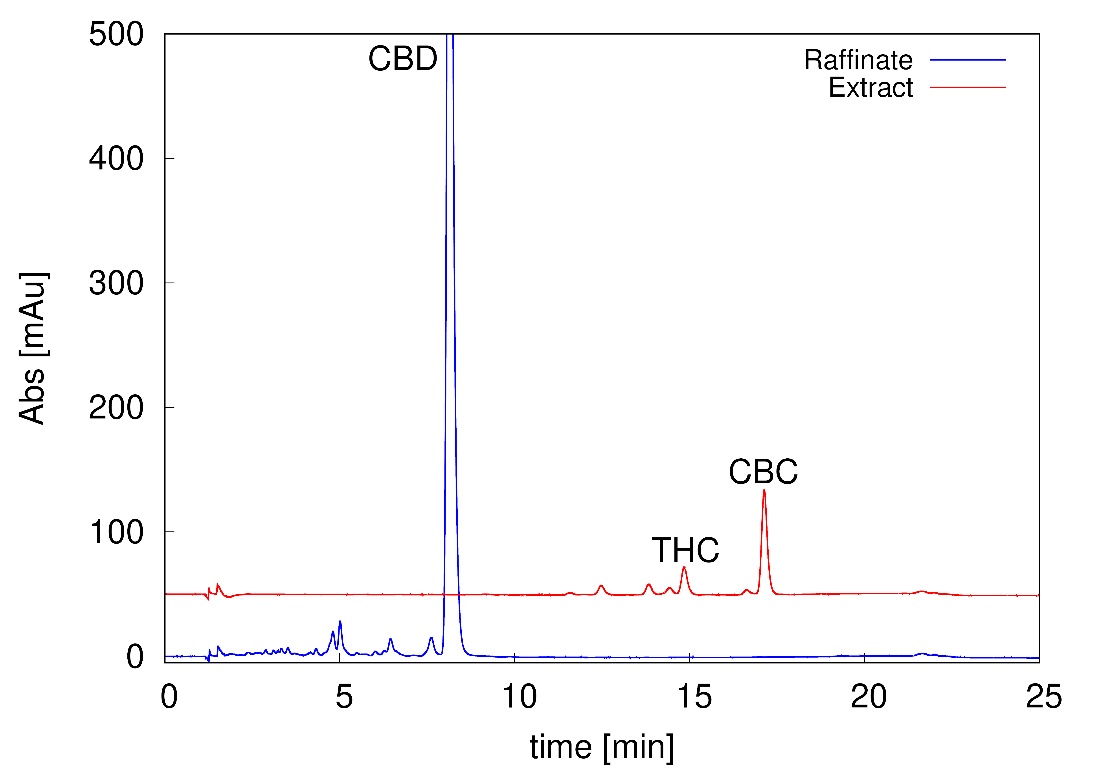
**

FIGURE S2: Analytical chromatograms of raffinate (blue) and extract (red) fractions obtained with gradient analytical method (see Sec. 4.2 of main text).

1. **AGREE PARAMETERS**

The eight parameters considered in this study for the calculation of the greenness of the two purification processes are the following:

1) **Positioning of analytical device**: indicating if the analytical system used to check purity and recovery of the purified fractions is located on-line (in = 1) or off-line the purification system (out = 0); since HPLC is used, a value of 0 was set for both batch and SMB.

2) **Degree of automation and continuous process**: automatic process indicates that minimal or no operator input are needed; continuous process indicates that feed is continuously injected and purified.

Automatic and Continuous=1, Automatic and Discontinuous=0.5, Manual and Discontinuous=0.

A value of 1 was assigned to SMB, since it is a completely automated and continuous system, and a value of 0.5 was assigned to batch, since the injection of the feed is discontinuous, but the system can be operated without operator intervention if the same method is subsequently run in a sequence.

3) **Solvent Consumption**: 0 ml/min = 1, 100 ml/min = 0; values of 0.97 and 0.59 were assigned to SMB (3.3 ml/mg) and batch (41 ml/mg), respectively.

4) **Productivity**: 100 mg/h = 1, 0 mg/h = 0; values of 0.6 and 0.06 were assigned to SMB (57 mg/h) and batch (6 mg/h), respectively.

5) **Purity**: 100% = 1, 0% = 0;

6) **Recovery**: 100% = 1, 0% = 0;

7) **Waste**: indicates if the purification system produces waste (yes = 0) or not (No = 1); values of 1 and 0 were assigned to SMB and batch, respectively.

8) **Solvent Toxicity**: indicates if the purification method uses toxic solvents, following solvents greenness scores^1^; green solvents = 1, toxic solvents = 0.

**REFERENCES**

1. L. Ferrazzano, M. Catani, A. Cavazzini, G. Martelli, D. Corbisiero, P. Cantelmi, T. Fantoni, A. Mattellone, C. De Luca, S. Felletti, W. Cabri, A. Tolomelli, Sustainability in peptide chemistry: current synthesis and purification technologies and future challenges, Green Chem. 24 (2022) 975.
